# Three cases of tubulointerstitial nephritis and uveitis syndrome with different clinical manifestations

**DOI:** 10.1007/s10792-016-0321-5

**Published:** 2016-08-10

**Authors:** Takamitsu Nagashima, Mami Ishihara, Etsuko Shibuya, Satoshi Nakamura, Nobuhisa Mizuki

**Affiliations:** Department of Ophthalmology and Visual Science, Yokohama City University Graduate School of Medicine, Yokohama, 236-0004 Japan

**Keywords:** Uveitis, Tubulointerstitial nephritis, Urinalysis, Urine β2-microglobulin, Urine *N*-acetyl-β-d-glucosaminidase

## Abstract

We here describe three different clinical manifestations of tubulointerstitial nephritis and uveitis (TINU) syndrome. We examined and diagnosed the following 3 patients: a 15-year-old boy with bilateral anterior uveitis (Case 1), a 14-year-old girl with bilateral papilledema (Case 2), and a 49-year-old woman with panuveitis (Case 3). The findings are presented herein. Case 1: The patient had bilateral anterior uveitis. Urinalysis revealed markedly increased β2-microglobulin and *N*-acetyl-β-d-glucosaminidase levels. As the patient was pathologically diagnosed with tubulointerstitial nephritis (TIN), we diagnosed TINU based on the presence of both uveitis and TIN. He was treated with oral corticosteroids. Case 2: This patient showed anterior uveitis and papilledema in both eyes. On initial examination, the urine test results did not show any abnormality. Three months later, high β2-microglobulin and *N*-acetyl-β-d-glucosaminidase levels were detected. As the patient was clinically diagnosed with TIN, we subsequently diagnosed TINU. Both the ocular and renal findings improved without treatment. Case 3: The patient developed bilateral panuveitis, retinal vasculitis, and macular edema, which were initially suspected to be sarcoidosis. However, she was pathologically diagnosed with TIN 12 months before the onset of uveitis; therefore, she was finally diagnosed with TINU. She recovered with local corticosteroid administration only. TINU may present with fundal features in addition to anterior uveitis. Detailed history taking and urinalysis are important to determine the presence of tubular disorders in similar patients.

## Introduction

Tubulointerstitial nephritis and uveitis syndrome (TINU) is a disease in which idiopathic acute tubulointerstitial nephritis (TIN) is complicated by uveitis. Since Dobrin et al. [1] first reported 2 cases of acute eosinophilic interstitial nephritis accompanied by anterior uveitis and myeloid sarcoma in 1975, at least several hundred cases have been reported. In Japan, 2 years after the report by Dobrin et al. and Kikkawa et al. [2] reported 2 cases of combined interstitial nephritis and uveitis that were not associated with myeloid sarcoma. The diagnostic criteria of TINU are defined as the presence of histopathologically confirmed acute TIN and uveitis, as well as the absence of systemic diseases (e.g., sarcoidosis) associated with these conditions. Patients who suffer from TINU typically develop acute-onset bilateral uveitis, which commonly becomes chronic or recurrent, and fever is the most common symptom of TINU in Japan [3]. Herein, we report 3 cases of TINU with different clinical manifestations.

## Case reports

### Case 1

A 15-year-old boy visited a neighborhood clinic for left eye pain and reduced visual acuity, and was diagnosed with left iridocyclitis. He also experienced flu-like symptoms. He was referred to our hospital for detailed examination and treatment on day 10 of the disease.

**Fig. 1:**
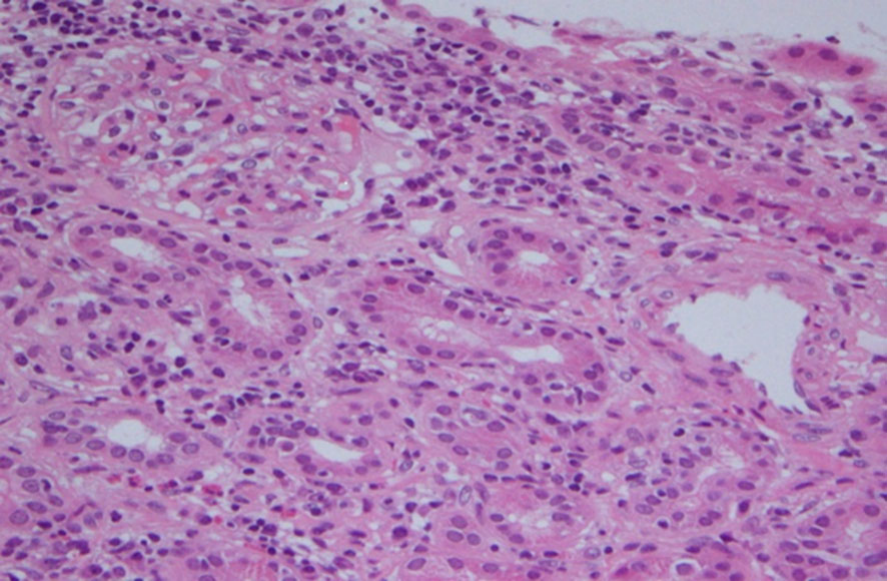
Pathological specimen obtained by renal biopsy (Case 1). Diffuse infiltration of inflammatory cells, mainly consisting of small lymphocytes, is observed in an area from the tubulointerstitium to the medulla

Among the initial findings, the corrected binocular visual acuity was 1.2. The intraocular pressures were 7 and 8 mmHg in the right and left eyes, respectively. He had 1+ inflammatory cells in the anterior chamber and fine keratic precipitates in both eyes. Iris synechiae and relative afferent pupillary defect (RAPD) were not observed. Mild diffuse vitreous opacity, redness, and swelling of the optic disk were detected in the left eye. Fluorescein angiography (FA) revealed leakage of fluorescent dye from the left optic disk, while neither vasculitis nor macular edema was observed. Blood tests revealed the following results: immunoglobulin G, 2175 mg/dL (normal range 870–1700 mg/dL); blood urea nitrogen, 20 mg/dL (10–15 mg/dL); creatinine, 1.63 mg/dL (0.6–1.2 mg/dL); erythrocyte sedimentation rate, 58 mm/h (≤10 mm/h); and C-reactive protein, 0.62 mg/dL (≤0.30 mg/dL). Urinalysis revealed the presence of protein and glucose, and β2-microglobulin (β2MG) and *N*-acetyl-β-d-glucosaminidase (NAG) levels of 30,295 μg/L (normal range ≤230 μg/L) and 26.8 U/L (normal range ≤7.0 U/L), respectively. The chest radiographic findings were normal, while abdominal plain computed tomography revealed mild enlargement of both kidneys. Renal biopsy revealed diffuse infiltration of inflammatory cells, mainly small lymphocytes, from the tubulointerstitium to the medulla (Fig. 1), leading to a pathological diagnosis of TIN. Based on the combination of uveitis and TIN, TINU was diagnosed.

The patient was treated with 1 course of steroid pulse therapy (methylprednisolone, 1000 mg/day for 3 days) and oral administration of prednisolone 30 mg, which was subsequently tapered. Around day 40, the anterior uveitis ameliorated and FA revealed no fluorescence leakage in the left optic disk. However, when the dose of oral prednisolone was tapered to 10 mg, anterior uveitis recurred along with an increase in the urine β2MG and NAG levels. Ocular and oral administration of steroids is being continued.

### Case 2

A 14-year-old girl visited a neighborhood clinic for right eye pain, and bilateral iridocyclitis was detected. She did not exhibit symptoms such as headaches or ringing in the ears. She was referred to our hospital for detailed examination and treatment on day 32 of the disease.

**Fig. 2:**
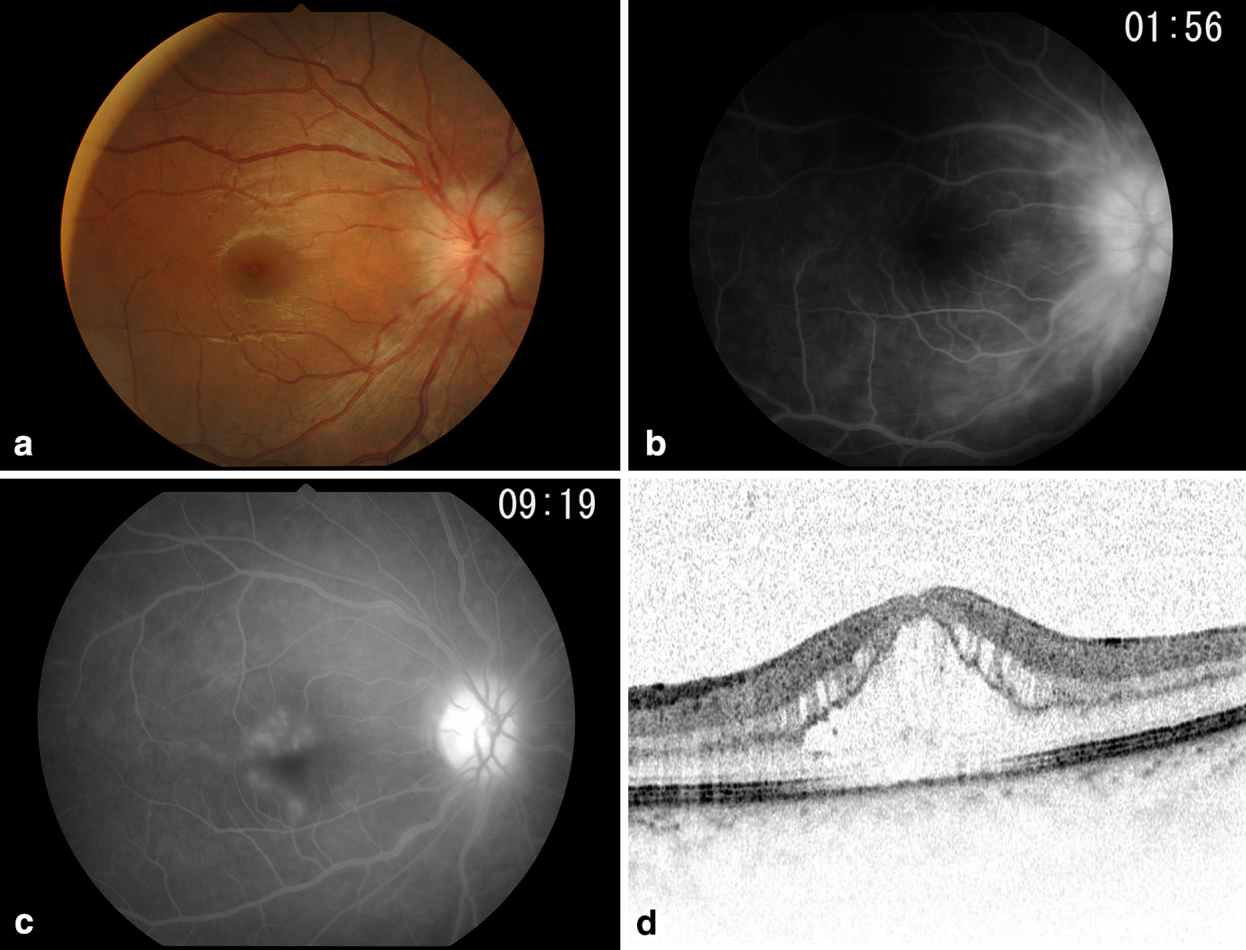
**a** Fundus photograph of the right eye (Case 2). Optic disk edema and vasculitis are observed. **b** Fluorescein angiography image of the right eye (Case 2). Leakage of fluorescent dye from the optic disk and retinal veins in the posterior pole is observed. **c**, **d** Fluorescein angiography and optical coherence tomography images (Case 3). The fluorescein angiogram revealed hyperfluorescence in the optic disk and leakage of fluorescent dye from the macula. Optical coherence tomography revealed retinal thickening and cyst-like changes in the macular area

The corrected binocular visual acuity was 1.2. The intraocular pressures were 19 and 20 mmHg in the right and left eyes, respectively. There was no inflammation in the anterior chamber, despite fine keratic precipitates being observed in both eyes. Iris synechiae and RAPD were not observed, whereas bilateral optic disk edema was detected (Fig. 2a). Goldmann perimetry revealed enlargement of Mariotte’s blind spot, and FA revealed leakage of fluorescent dye from the optic disks and retinal veins in both eyes (Fig. 2b). However, computed tomography did not reveal swelling of the optic nerve. The critical fusion frequency value was within the normal range in both eyes.

At the initial visit, neither the blood test nor urinalysis revealed any abnormality. No increase in the angiotensin-converting enzyme level was observed, and there was no sign of bilateral hilar lymphadenopathy on chest radiography. Three months later, urinalysis showed positive protein and glucose findings, and β2MG and NAG levels of 1210 μg/L and 12.9 U/L, respectively.

Since the patient had a cat, cat-scratch disease was suspected. However, she was negative for the anti-Bartonella antibody. TIN was clinically diagnosed without renal biopsy, and TINU was diagnosed based on the combination of uveitis and TIN. On day 81, the right papilledema improved after a triamcinolone acetonide injection in the sub-Tenon’s capsule. Around day 150, despite worsening of the left papilledema, treatment with only ocular steroid administration was continued. Around day 240, the renal dysfunction resolved spontaneously, and the left papilledema was simultaneously relieved. During the disease course, no eye depigmentation, nummular chorioretinal scars, retinal pigment epithelium aggregation, or migration was observed.

### Case 3

A 49-year-old woman visited a neighborhood clinic for right eye pain and was diagnosed with iridocyclitis. Subsequently, she was referred to our hospital for detailed examination and treatment on day 18 of the disease.

The corrected binocular visual acuity was 1.2. The intraocular pressure was 15 mmHg in both eyes. The right anterior chamber had 2+ inflammatory cells, moderate-to-small keratic precipitates, and peripheral anterior synechiae in both eyes. Optic disk edema and macular edema were detected in the right eye. FA revealed hyperfluorescence in the right optic disk, cystoid macular edema, and leakage of fluorescent dye from the peripheral retinal veins (Fig. 2c, d). The patient had been pathologically diagnosed with TIN at another hospital 12 months earlier, and underwent oral prednisolone treatment, which was tapered from 30 mg. Upon admission, blood test and urinalysis revealed only a mild increase in the serum creatinine.

Whole-body imaging studies were performed for suspected sarcoidosis, but the findings did not support this diagnosis. Instead, based on the presence of both uveitis and TIN, TINU was diagnosed. The right anterior uveitis and macular edema were relieved by ocular steroid administration. Around day 180, inflammation of the anterior segment and macular edema occurred in the left eye, but these symptoms were also relieved by ocular steroid administration.

## Discussion

We observed 3 cases of TINU with different clinical manifestations (Table 1). Although this disease can occur in all ages and sexes, it is more likely to occur in peripubertal girls (median age, 15 years; male-to-female ratio, 1:3) [3]. TINU reportedly accounts for 1–2% of uveitis cases in children aged ≤15 years [4]. There are also reports of TINU in middle-aged people [5, 6], which are similar to Case 3 herein. Matsumoto et al. summarized the findings from 102 Japanese patients with TINU that were reported between 1977 and 2013 [5]. In their study, TINU showed a female predominance (approximately 2.5:1) and was more likely to develop in younger patients, with a median age of 14 years. Slight fever was the most common general symptom. Similarly, in Case 1, flu-like symptoms were some of the first clinical symptoms.

**Table 1:** Comparison of three cases of tubulointerstitial nephritis and uveitis

	Case 1	Case 2	Case 3
Age (years)/sex	15/male	14/female	49/female
Onset of ocular/renal manifestations	Concurrent onset	Uveitis preceded manifestations	TIN^a^ preceded manifestations
Inflammation of the anterior segment	Non-granulomatous	Non-granulomatous	Granulomatous
Fundus features	Diffuse vitreous opacity	Papilledema, retinal vasculitis	Macular edema, retinal vasculitis
FA^b^ findings (optic disc)	Hyperfluorescence	Leakage of fluorescent dye	Hyperfluorescence
FA findings (retinal vessels)	None	Leakage of fluorescent dye from the retinal vessels in the posterior pole	Leakage of fluorescent dye from the peripheral retinal vessels
Differential diagnosis		Cat scratch disease	Sarcoidosis
Treatment for nephritis	Steroid pulse therapy	No treatment	Oral administration of steroids
Course of renal dysfunction	Recurrence	Spontaneous remission	No recurrence
Course of uveitis	Recurrence	No recurrence	Recurrence

^a^ *TIN* tubulointerstitial nephritis

^b^ *FA* fluorescein angiography

Uveitis reportedly develops between 2 months prior to 12 months after the onset of TIN, occurring before, simultaneously, and after the diagnosis of TIN in 20, 15, and 65 % of cases, respectively [3]. Although cases of concurrent onset of uveitis/TIN (Case 1) are easy to diagnose, cases in which uveitis occurs after nephritis (Case 3) require differentiation from other diseases [7]. The differential diagnoses of TINU include systemic lupus erythematosus, sarcoidosis, Sjögren’s syndrome, Behcet’s disease, tuberculosis, toxoplasmosis, and infectious mononucleosis. In Case 3, differentiation from sarcoidosis complicated by granulomatous uveitis and renal lesions was important. If the history of TIN had not been confirmed, the diagnosis might not have been established. Importantly, it should be noted that uveitis tends to become chronic in middle-aged patients [8]. Moreover, in cases of uveitis preceding TIN [9], such as Case 2, it is important to repeat whole-body imaging studies in order to differentiate from other diseases. However, in some cases, the nephritis is mild and resolves spontaneously, which makes it difficult to diagnose [10].

In this study, Cases 1 and 2 presented with non-granulomatous uveitis, while Case 3 presented with granulomatous uveitis. Although non-granulomatous anterior uveitis associated with fine keratic precipitates is typically observed in TINU, recurring/prolonged cases may be accompanied by mutton-fat keratic precipitates (granulomatous inflammation), fibrin deposition, posterior synechiae, hypopyon, etc. [11]. The reported features of the fundus in TINU include papilledema, tortuosity, and dilatation of the retinal vessels in the posterior pole, peripheral exudates, chorioretinal scars [3, 12, 13], and concurrent choroidal neovascularization [14]. Moreover, neuroretinitis may be exhibited in TINU [9]. A common FA finding of TINU is the leakage of fluorescent dye from the optic disks and central-to-peripheral microvessels, which was observed in our cases [15].

In Case 2, leakage of fluorescent dye from the optic disk and optic disk edema on optical coherence tomography were observed, and Goldmann perimetry revealed enlargement of Mariotte’s blind spot. Therefore, differential diagnoses of optic neuritis and uveitis with optic disk edema were considered.

However, as RAPD was not recognized, and no swelling of the optic nerve was observed upon computed tomography, we excluded optic neuritis. Moreover, the critical fusion frequency value in both eyes was within the normal range. We also considered the possibility of a disease related to intercurrent optic disk edema and uveitis, such as sarcoidosis, Vogt–Koyanagi–Harada disease, or cat-scratch disease. However, this case exhibited non-granulomatous uveitis, and sarcoidosis and Vogt–Koyanagi–Harada disease are both classified as granulomatous uveitis. Therefore, we considered that these diseases were unlikely in this patient. Moreover, in this case, vasculitis was seen upon FA, while the distinctive periphlebitis with perivascular nodules associated with sarcoidosis was not recognized. In addition, no increase in the angiotensin-converting enzyme level was observed in the biochemical tests, and there was no bilateral hilar lymphadenopathy upon chest X-ray, excluding a diagnosis of sarcoidosis. Concerning the differential diagnosis of Vogt–Koyanagi–Harada disease, a type of optic neuritis that does not exhibit serous retinal detachment, symptoms of meningitis, including headache, were not observed, nor were the prodromes of Vogt–Koyanagi–Harada disease, including ringing in the ears and dizziness. Further, this case showed a self-limiting trend without systemic steroid administration, and ocular depigmentation, nummular chorioretinal scars, and retinal pigment epithelium clumping and migration were not observed, thus ruling out Vogt–Koyanagi–Harada disease. Finally, cat-scratch disease remained as a potential differential diagnosis; however, no anti-Bartonella antibodies were detected and a “star figure” did not appear in the macula even over time, suggesting that this was not the correct diagnosis. Instead, from the above findings, we eventually diagnosed this case as TINU. It should also be noted that the positive predictive value of increased β2M and serum creatinine has been reported to be 100 % for detecting TINU [16]. Additionally, NAG is a good screening marker for detecting renal dysfunction in TINU [17]. However, while Case 1 showed increased urinary β2M and serum creatinine levels, Case 2 showed only increased urinary β2M levels.

Matsumoto et al. [5] reported that, in Japanese patients with TINU, the efficacy of systemic corticosteroids was unclear, while ocular administration of steroids to treat uveitis was effective in 60 % of patients. If systemic administration of steroids shows poor efficacy in the treatment of TINU, administration of an immunosuppressive agent such as methotrexate or cyclophosphamide should be considered. Methotrexate is considered to be very useful in the tapering of steroids; however, it is associated with severe side effects such as bone marrow suppression, interstitial pneumonia, and stomatitis. On the other hand, although cyclosporine is associated with a risk of renal toxicity, it is often used for steroid-resistant cases of non-infectious uveitis in Japan. In Case 1, when the dose of oral prednisolone was tapered, anterior uveitis recurred along with an increase in the urine β2MG and NAG levels. However, although the patient experienced relapse of symptoms during steroid tapering, the efficacy of the steroids at the initial dose was good, and cyclosporine administration was therefore not needed in this case [18, 19]. On the other hand, in Cases 2 and 3, systemic corticosteroids were not needed.

Typical pathological features of TIN include infiltration of inflammatory cells (predominantly lymphocytes along with neutrophils and plasmatocytes) to the tubulointerstitium. Edema and atrophy of tubular epithelial cells may also be observed. Conversely, the normal structure of the glomerular matrix, blood vessels, and the number of mesangial cells are maintained. Lastly, using fluorescent antibodies, deposition of immunoglobulins and complements may be observed. In the present study, the pathological features of Case 1 were consistent with these findings, whereas pathological testing was not performed and was performed at another hospital for Cases 2 and 3, respectively; hence, the pathological features of these patients could not be verified.

Moreover, in our cases, we did not examine the human leukocyte antigen (HLA) types. A genetic background has been suggested in Caucasian patients with TINU (associations of HLA-A2 and -A24 with TINU) [3], but no specific HLA types related to TINU have been identified in Japanese patients [5].

Finally, TINU is known to have a relatively favorable prognosis, while there are some reports of poor visual prognosis, such as in cases with choroidal neovascularization [14]. Thus, early diagnosis and treatment are recommended.

In conclusion, when a diagnosis of TINU is considered, detailed history taking along with whole-body imaging studies and examinations of renal function are important.
